# *Loa loa* vectors *Chrysops* spp.: perspectives on research, distribution, bionomics, and implications for elimination of lymphatic filariasis and onchocerciasis

**DOI:** 10.1186/s13071-017-2103-y

**Published:** 2017-04-05

**Authors:** Louise Kelly-Hope, Rossely Paulo, Brent Thomas, Miguel Brito, Thomas R. Unnasch, David Molyneux

**Affiliations:** 1 Department of Parasitology, Liverpool School of Tropical Medicine, Liverpool, UK; 2 CISA, Health Research Centre of Angola, Caxito, Angola; 3 Lisbon School of Health Technology, Lisbon, Portugal; 4 College of Public Health, Department of Global Health, University of South Florida, Tampa, USA

**Keywords:** *Loa loa*, Loiasis, Tropical eye worm, *Chrysops*, Vector control, Lymphatic filariasis, Onchocerciasis, Neglected tropical diseases, NTDs, Africa, Integrated vector management, Bionomics

## Abstract

**Background:**

Loiasis is a filarial disease caused *Loa loa*. The main vectors are *Chrysops silacea* and C*. dimidiata* which are confined to the tropical rainforests of Central and West Africa. Loiasis is a mild disease, but individuals with high microfilaria loads may suffer from severe adverse events if treated with ivermectin during mass drug administration campaigns for the elimination of lymphatic filariasis and onchocerciasis. This poses significant challenges for elimination programmes and alternative interventions are required in *L. loa* co-endemic areas. The control of *Chrysops* has not been considered as a viable cost-effective intervention; we reviewed the current knowledge of *Chrysops* vectors to assess the potential for control as well as identified areas for future research.

**Results:**

We identified 89 primary published documents on the two main *L. loa* vectors *C. silacea* and *C dimidiata*. These were collated into a database summarising the publication, field and laboratory procedures, species distributions, ecology, habitats and methods of vector control. The majority of articles were from the 1950–1960s. Field studies conducted in Cameroon, Democratic Republic of Congo, Equatorial Guinea, Nigeria and Sudan highlighted that *C. silacea* is the most important and widespread vector. This species breeds in muddy streams or swampy areas of forests or plantations, descends from forest canopies to feed on humans during the day, is more readily adapted to human dwellings and attracted to wood fires. Main vector targeted measures proposed to impact on *L. loa* transmission included personal repellents, household screening, indoor residual spraying, community-based environmental management, adulticiding and larviciding.

**Conclusions:**

This is the first comprehensive review of the major *L. loa* vectors for several decades. It highlights key vector transmission characteristics that may be targeted for vector control providing insights into the potential for integrated vector management, with multiple diseases being targeted simultaneously, with shared human and financial resources and multiple impact. Integrated vector management programmes for filarial infections, especially in low transmission areas of onchocerciasis, require innovative approaches and alternative strategies if the elimination targets established by the World Health Organization are to be achieved.

**Electronic supplementary material:**

The online version of this article (doi:10.1186/s13071-017-2103-y) contains supplementary material, which is available to authorized users.

## Background

Loiasis - also known as Tropical eye worm, is a filarial disease caused by *Loa loa*, a parasite which mainly occurs in Central and West African rainforests [1, 2]. *Loa loa* is transmitted by two main species of tabanid flies (Order Diptera: Family Tabanidae) of the genus *Chrysops*, and include *Chrysops silacea* (Austen) and *C. dimidiata* (Wulp), which are forest canopy dwellers. The distribution of loiasis has recently been well documented and mapped from large-scale community field surveys based on the presence of eye worm [2, 3], and defined earlier by remote sensing maps of forest and forest edges [4]. The risk of loiasis geographically coincides with the boundaries of equatorial rainforest, with the tropical dense and mosaic savanna forests (outside the Congo River Basin) shown to be important determinants of *L. loa* as they are natural habitats of the main *Chrysops* spp. [2, 5].

Loiasis symptoms are considered to be relatively mild but include itching and swelling as the worm moves under the skin and causes lesions, typically in the extremities, called Calabar swellings and the passage of the adult worm in the sub-conjunctiva of the eye [1]. However, the real danger of loiasis occurs when an infected person with high levels of *L. loa* microfilariae (Mf) in their blood (>30,000 Mf/ml) take the drug ivermectin or diethylcarbamazine (DEC) for the treatment of lymphatic filariasis (LF) or onchocerciasis. These individuals are at increased risk of a severe adverse event (SAE), which may result in encephalopathy and death [6, 7]. A recent cohort study has also found an increased risk in mortality among individuals with a high Mf loads of *L. loa* [8].

Severe adverse events were first documented during ivermectin distribution projects in Cameroon in the early stages of the African Programme for Onchocerciasis Control (APOC) when the community directed treatment with ivermectin (CDTi) was the main intervention. Later SAEs were also recorded in the Democratic Republic of Congo (DRC), and SAEs have had significant negative repercussions for the onchocerciasis programmes over the past two decades reducing the opportunities to expand ivermectin distribution and reducing adherence to mass drug administration. The threat of SAEs have prevented the Global Programme to Eliminate LF (GPELF) scaling up mass drug administration (MDA), as ivermectin was considered unacceptable given the associated risks, and an alternative strategy of twice a year albendazole was recommended where LF and *L. loa* were co-endemic. As both the LF and onchocerciasis programmes have defined elimination objectives the problem of *L. loa* associated SAE risk must be resolved if elimination is to be achieved.

In *L. loa* co-endemic areas, the LF Programme has an advantage as the main vectors are *Anopheles* spp. and malaria control measures are known to impact on the transmission of *Wuchereria bancrofti* parasite, in particular indoor residual spraying (IRS) and bed nets or long-lasting insecticidal nets (LLINs) impregnated with pyrethroids [9–11]. However, the major challenge lies with onchocerciasis, now targeted for elimination and which now includes treating low transmission areas, previously described as “hypo-endemic” and not included in the APOC programme as the disease was not considered to be a major public health problem. The method of determination of the endemicity of onchocerciasis to be eligible for MDA with ivermectin was based on the prevalence of nodules in small samples of adults (50), and if found to be less than 20% it was considered no MDA was necessary as the area was defined as “hypo-endemic”. The extent of the areas of low transmission of *Onchocerca volvulus* have been identified, and mapping the risk of *L. loa* in these areas determined. This has helped to identify a number of areas of highest risk of *L. loa-*associated SAEs, which have been referred to as ‘hypo-endemic hotspots’, and will help country programmes and partners to plan locally the defined interventions necessary [12].

The use of this information for both the LF and onchocerciasis programmes is a prerequisite for effective programmatic success if the ever persistent problem of loiasis is to be addressed by programmes, and the elimination of LF and onchocerciasis is to become a reality [13]. The epidemiological complexity of these problems has been highlighted by Molyneux et al. [13], and more recently by the observations that there is cross-reactivity of the rapid antigen diagnostic BinaxNOW Filariasis immunochromatographic test (ICT), where positive ICT positive cases have been shown to be the result of infection with *L. loa*, thus complicating the diagnostic and monitoring assessments required of LF programmes [14–17].

To date the control of the *Chrysops* vector of *L. loa* has not been considered as a potential alternative or additional strategy to address the problem co-endemic loiasis presents to the LF and onchocerciasis elimination programmes. It is possible it could play an important role if correct strategies are deployed. However, a better understanding of the major vectors transmitting *L. loa* is essential and timely given the World Health Organization (WHO) defined Roadmap targets for the elimination of LF and onchocerciasis, and the challenges identified [18]. The aim of this review, is to collect and synthesise the current knowledge of the distribution of the two main vectors *C. silacea* and *C. dimidiata*, highlighting main field and laboratory procedures, species distributions, ecology, habitats, potential methods of vector control and areas for future research, which may have implications for the filariasis elimination programmes in a significant part of Africa.

## Methods

A systematic search and collation of data in the peer-reviewed published literature on the two main *Chrysops* spp. of vectors of *L. loa* was conducted using PubMed, JSTOR, SCOPUS and Google online sources. Search terms, and combinations thereof, included *Loa loa*, *L. loa*, loiasis, Rapid Assessment Procedure for Loiasis (RAPLOA), *Chrysops*, *C. silacea* and *C. dimidiata*, Tabanid, Africa. All published literature with information on the two main *Chrysops* vector species, was reviewed. Information on other secondary vectors were documented where appropriate to provide perspective on the different potential vectors; however, they were not the focus of the review. Further references were obtained from the references listed within articles, and from the references within those articles and so on. Articles that were not obtainable through online sources were sourced through the Liverpool School of Tropical Medicine Library where possible. Information on the articles were collated into a database in Excel (Microsoft) (Additional file 1). The following information was summarised:

- **Publication profile** including (i) number of articles; (ii) time of publication (year and decade); (iii) type of article (research, review, thesis, report); (iv) journal/ publisher (name); and (v) institution (name and location; based on lead author’s affiliation);
- **Study features** including (i) country and locality; (ii) type of study (field, laboratory, field/laboratory); and (iii) study period (start and duration);
- **Field and laboratory procedures** including (i) collection methods (adult and immature stages of *Chrysops*); (ii) species identification; and (iii) infection detection;
- **Species distribution, ecology and habitats** including (i) distribution and ecology; (ii) immature stage habitats; (iii) adult habitats; (iii) host seeking patterns; (iv) host preference; and (v) flight range;
- **Factors influencing spatial-temporal transmission** including (i) abundance patterns (daily, monthly seasonal); (ii) spatial environmental factors; and (iii) temporal environmental factors, anthropogenic factors (plantations, wood fire);
- **Methods of vector control** including (i) defensive control measures (screening, repellents, clearing forest and bush); and ii) aggressive control measures (insecticide larvicides, adulticides).

Information on the study locations included in the published documents were geo-referenced and imported into the geographical information system software ArcGIS 10.1 (ESRI, Redlands, CA) to produce a new vector distribution map based on the knowledge synthesised in this review.

Based on the information reviewed, key points related to field and laboratory procedures, species distribution, ecology and habitats, spatial-temporal transmission and methods of vector control were highlighted in a series of excerpts, and areas for potential future research were summarised.

## Results

### Publication profile

**Fig. 1:**
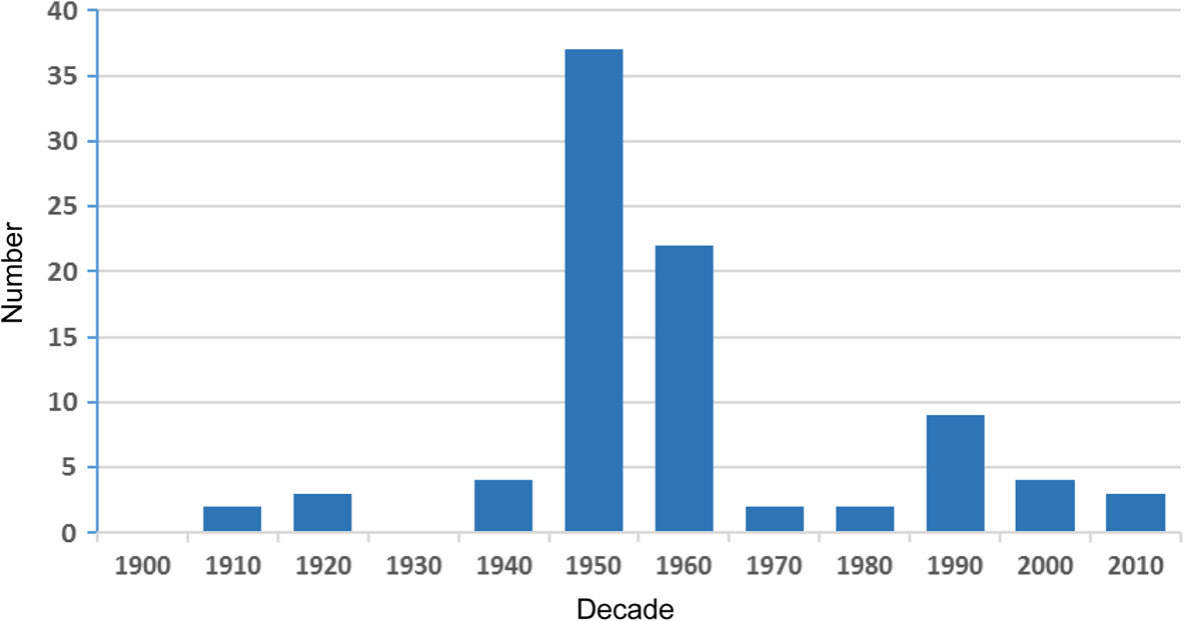
Number of articles per decade 1900–2010

In total, 89 published documents with information on the two main *L. loa* vectors *C. silacea* and *C dimidiata* were collated into a database (see Additional file 1) [19–103]. The number of articles published per decade ranged from 0 to 37, with the highest number published in the 1950s (Fig. 1). The majority of articles were research based (*n* = 68) with several related reviews or combinations of research/ review (*n* = 18), one book chapter, conference abstract, and one PhD thesis by Crewe in 1956 [57]. The three most extensive reviews were published over 50 years ago by Gordon et al. 1950 [28], as part of the ‘Symposium on Loiasis’ in 1955 [47] and in book chapters by Oldroyd [61], while two briefer, more general reviews, were published in decades thereafter [84, 89], More than half of the research articles were part of a series of interlinking studies and include the following:

(i) ‘Observations on *Chrysops silacea* and *C. dimidiata* at Benin, southern Nigeria’ by Davey and O’Rourke published in 1951 (three articles) [30–32];
(ii) ‘Studies on the intake of microfilaria by their insect vectors, their survival and their effect on the survival of their vectors’ by Kershaw and Duke between 1951 and 1954 (six of ten articles) [38, 40, 41, 44, 59, 60];
(iii) ‘Studies on the epidemiology of filariasis in West Africa, with special reference to the British Cameroons and the Niger Delta by Kershaw and Nicholas between 1950 and 1955 (three of six articles) [29, 39, 45];
(iv) ‘Studies on the biting habits of *Chrysops*’ by Duke between 1955 and 1959 (seven articles) [50–56];
(v) ‘Studies on the control of the vectors of loiasis in West Africa’ by W. Crewe and P. Williams between 1962 and 1964 (eight of nine articles) [75–83];
(vi) ‘Studies of Ethiopian *Chrysops* as possible vectors of loiasis’ by W. Crewe and P. Williams published between 1954 and 1960 (three articles) [42, 63, 64];
(vii) ‘The bionomics of the tabanid fauna of streams in the rain-forest of the Southern Cameroons published by W. Crewe and P. Williams between 1961 and 1962 (four articles) [68–71].

The majority of articles were published in the Annals of Tropical Medicine and Parasitology (*n* = 45): active between 1907 and 2012 and now known as Pathogens and Global Health, and the Transactions of the Royal Society of Tropical Medicine and Hygiene (*n* = 13; active since 1907), two major journals still publishing today. There were other journals that published papers on *Chrysops* spp. from Belgium, Egypt, France, Germany, Pakistan, UK and Zimbabwe, the details are found in the Additional file 1. Based on the lead author’s affiliation, the majority of the research was undertaken by universities or research centres.

The majority of articles were from researchers based at the Helminthiasis Research Scheme, Kumba, British Cameroons (now in Cameroon), which was set up specifically on the recommendation of the Colonial Medical Research Committee to study loiasis with collaborating partners from the University of Liverpool and/or the Liverpool School of Tropical Medicine, UK, and collectively account for more than half the studies published. It was recognised that in order to control loiasis, a better understanding of the *Chrysops* spp. vectors driving transmission was required [66].

### Study features: location, type and period

The majority of research studies were conducted in Cameroon in the surrounds of Kumba and Bombe villages in an area formerly known as British Cameroons in the south western region of the country (*n* = 48), and close to where the Helminthiasis Research Scheme was based. Other research studies were conducted in Nigeria (southern States: Cross River, Oyo, Ogun, Ondo), Congo (Chaillu Mountains), DRC (nationwide), Equatorial Guinea (Bioko Island), Gabon (Reserve Ipassa-IRET Makokou) and Sudan (southern region). The most common type of study was field-based (*n* = 30) or a combination of field/ laboratory-based (*n* = 28) with only a few laboratory-based studies (*n* = 6). Overall, information on the study period was irregular with the year the study started most regularly documented. More specific information on the exact month, season and duration of studies were less well documented.

### Field and laboratory procedures

#### Collection methods

All field-based studies involved outdoor collections either of adult or immature-stage/ larval stages and were mainly related to measuring transmission patterns including species abundance and infection rates (Additional file 1). The main method of collecting adult *Chrysops* spp. was the use of local men (historically known as “fly-boys”), with hand nets to capture the host-seeking fly, which once caught were secured in containers or test tubes for quantification or further analysis in the laboratory.

**Adult collection method** *Each fly-boy was armed with a small hand-net made of mosquito-netting, about 6 inches in diameter and a short handle about 12 inches long, and with a test-tube. … or each team of boys had one Barraud cage in which to keep the catch … sat down and caught flies that came to feed on him … transferring to them to the cage. (Kumba, Cameroon)*

The immature stages of *Chrysops* were collected using a simple apparatus built to sieve mud from shallow streams or swampy areas to identify larvae and pupae. Historical photographs of the field apparatus are shown in Additional file 2 [47, 57].

**Immature-stage/larvae and pupae collection method** *… it consisted of a wooded-framed sieve 16 inches square and 2 inches deep mounted on four legs to form a table 30 inches high; ordinary mosquito-screening wire is used for the active sieve. On top of the “table” rests a similar sieve without legs and a ¼ inch square mesh. The table is fixed in a suitable position, usually standing in the stream and mud from the breeding site is placed on the upper coarse sieve and washed through with water. Large pieces of debris, sticks and stones are retained by the coarse sieve, which is then removed. The mud is then slowly washed through the fine sieve and the larvae and pupae collected as they become visible. (Kumba, Cameroon)*

#### Species identification

**Fig. 2:**
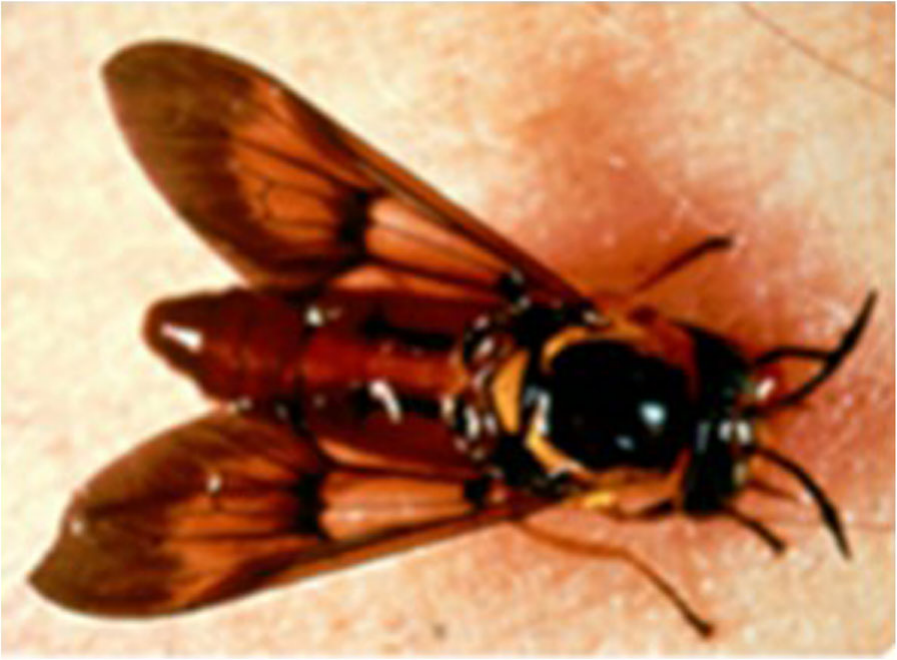
Picture of *Chrysops silacea*. Source: https://www.cdc.gov/parasites/loiasis/

Information on species identification were not commonly documented, however, from the articles published, both *C. silacea* and *C. dimidiata* have only been identified and distinguished from each other by morphological features [54, 61]. Overall, the two species are similar with a characteristic colour, longitudinal black stripes on abdomen, mottled wings and large head and eye (Fig. 2). In some parts of West Africa, *C. silacea* is known as the ‘Red Fly’ [61, 66, 102] due to its bright orange abdomen with short black stripes, which was considered distinct from *C. dimidiata* with its paler colour and broader longer stripes. Field workers were found to have no problem distinguishing them apart with noted typical ‘silacea’ and ‘dimidiata’ characteristics [21, 23, 66].

#### Infection detection

*Loa loa* were documented to be found in the fat-body of abdomen and to a lesser extent the fat-body of the thorax and head of *Chrysops* spp. *Loa loa* larvae were classified into different stages including sausage (L1), larval stage 2 (L2) and larval stage 3 or infective stage (L3), with the development of microfilariae to the infective stage estimated to take between 10 and 12 days based on laboratory experiments [22, 23]. Dissecting *Chrysops* spp. under a microscope was the only method used for detecting infection, which involved separating the head, thorax and abdomen manually, and identifying the presence (parous) or absence (nulliparous) of *L. loa* larva [56, 99]. Transmission was related to the frequency of L3 found in the head of the flies and the biting density of vectors with the main measures including (i) parous rates (PR) estimated as the proportion of parous flies to the total number dissected; (ii) potential infection rates (PIR) estimated as proportion of flies with L3s; (iii) infective rates (IR) determined as the proportion of flies with L3s in the head [90, 99, 103].

### Species distribution, ecology and habitats

#### Distribution and ecology

**Fig. 3:**
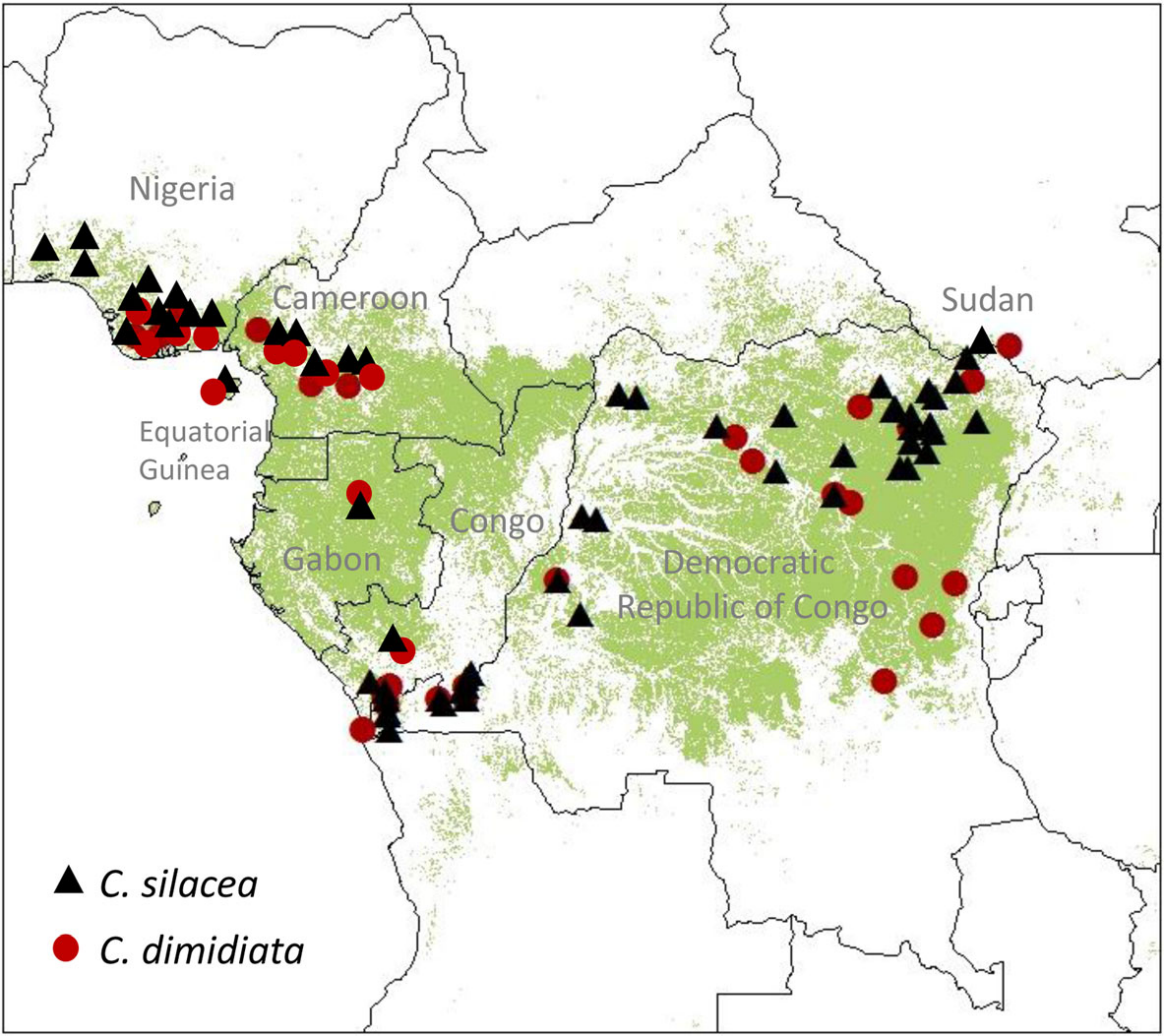
Map showing reported species distribution

The broad distributions of the main vectors, *C. silacea* and *C. dimidiata* are shown in maps (Fig. 3), which were based on available georeferenced data of study locations and four historical maps (see Additional file 3). Overall *C. silacea* and *C. dimidiata* have been found throughout the greater part of the tropical equatorial rainforest. They are considered to become less dominant on the fringes where other species may replace them as vectors, as seen in southern Sudan and Central Nigeria where *C. distinctipennis* is the dominant savanna species, and well known to local inhabitants [24, 46]. Additional forest species include *C. langi* and *C. centurionis*, while *C. zahrai* is a forest-fringe species and *C. longicornis* both a forest and savanna species [61]. However, these additional species were not considered to be primary vectors of human *L. loa*, and more associated with maintaining the monkey ‘strain’ of *L. loa* through crepuscular biting and nocturnal periodicity. They were reported to be reluctant to feed on humans; however, *C. zahrai* was reported to feed on humans if they are out in the forest after dark during peak biting time of this species. Table 1 summarises key characteristics of the different species in relation to habitat, host, and periodicity [46, 47, 73].

**Table 1:** Summary of primary and secondary *Chrysops* spp. main characteristics

Species	Ecological distribution	Peak biting time	Putative host	Main biting location
*C. silacea*	Forest	Day	Human	Ground
*C. dimidiata*	Forest	Day	Human	Ground
*C. langi*	Forest	Crepuscular/Nocturnal	Monkey	Canopy
*C. centurionis*	Forest	Crepuscular/Nocturnal	Monkey	Canopy
*C. zahrai*	Forest-fringe	Crepuscular	Monkey/Human	Canopy/Ground
*C. longicornis*	Forest/Savanna/Wooded areas	Crepuscular	Monkey	Canopy
*C. distinctipennis*	Savanna	Crepuscular	Monkey/Human	Canopy/Ground

Overall, *C. silacea* and *C. dimidiata* were considered to have similar habitats, and in addition to rainforests, have been found in rubber plantations, palm oil groves and fringes of mangrove swamps [32]. Both species frequently occur together; however, in some areas one species was found to dominate the other, and across different ecological settings with *C. silacea* more likely to adapt to human influenced environments. For example, *C. silacea* was reported to be more abundant in Kumba, Cameroon (rainforest), Sapele, Nigeria (rubber plantation) and Congo (rainforest) [91]; however, the latter author noted that *C. dimidiata* was more abundant in the palm groves within the forested study area. *Chrysops dimidiata* was reported to be more abundant in Benin, Nigeria (palm grove) [30]; Eseka in central Cameroon (rainforest) [61], Bioko island, Equatorial Guinea (rainforest) [100], and in Akamkpa Community, Cross Rivers State, Nigeria (rainforest); however, for the latter it was noted that *C. silacea* was more abundant in the adjacent mangrove forest [102].

#### Immature stage habitats

The *Chrysops* larvae and pupae were found to have well defined microhabitats, which were characterised by densely shaded streams and swamps, shallow slow flowing or standing water, with fine soft mud covered by layers of decaying leaves [28, 32, 57]. These habitats were noted be markedly acidic probably due to the decaying organic matter. *Chrysops* larvae were also reported in the streams draining the borders of a rubber plantations into the surrounding mangrove swamps. Photographs of typical breeding sites are shown in Crewe [57], and Gordon et al. [26] available in Additional file 2. In Benin (Nigeria), extensive larval habitat studies where *C. dimidiata* was the dominant vector, showed larvae were predominately found in less than three inches of mud, and in areas of saturated or damp mud but not where water was one foot, or mud more than three inches in depth [26, 32].

***Chrysops silacea*** **forested larval breeding site** *Chrysops at Kumba considered very restricted, and confined to certain habitats in densely shaded, where slowly moving water passes over a layer of mud covered in decaying vegetation. Generally, the thickly overgrown valleys flanking the residential areas have densely shaded streams at the bottom, and in parts the streams are impeded by vegetation, making the water slow, and the bottom is covered by fine sand overlaid with soft mud which is covered in decaying leaves and considered Chrysops breeding places (Kumba, Cameroon)*.

***Chrysops dimidiata*** **plantation larval breeding site** *… breeding was confined to certain reaches of the river: where banks were swampy and where there was a thick mass of decaying vegetable matter over mulch, larvae were common, but where the edges of the river were clear-cut and sandy, and thus devoid of organic matter, no specimens were ever taken. (Benin, Nigeria)*

#### Adult habitats

*Chrysops silacea* and *C. dimidiata* were considered to be forest canopy dwellers descending to bite the human population in the forested or plantation areas. *Chrysops silacea* in particular has been reported to avoid the deepest shade and the brightest sunlight, and found to be most abundant in the patchy light-shade of intermediate areas [47]. This vector has been found to bite at all levels of the forested areas, and throughout plantations, and will leave shelter to cross small clearings to enter houses or attack local workers. In Sapele, Nigeria, the rubber plantations bounded by swamps were considered to provide exclusive contact sites between human and flies, with no competing hosts. This appeared to lead to a different transmission pattern with many labourers infected, a high abundance of *Chrysops* and high levels of infection in local *Chrysops* populations [21, 32, 61].

**Rubber plantation (predominately** ***Chrysops silacea*** **)** *The rubber trees are mature … about 50 feet high. The branches are interlaced, and form a continuous thick canopy, which casts a deep shade through which little direct sunlight penetrates … There is no monkey population in the canopy, and the attention of the flies are concentrated upon the African rubber-tappers. Moving about their duties, and clearly visible from above. (Sapele, Nigeria)*

#### Adult host-seeking

*Chrysops silacea* and *C. dimidiata* were considered to be practically noiseless, persistent daylight feeders attacking the ankles and the lower limbs most commonly [31, 57]. They were considered to hunt mainly by sight and noted to be attracted to colour and movement; however, specific studies on host seeking behaviour also found an olfactory stimulus related to forest leaves burning in wood fires [51] this attraction to fires perhaps due to the CO2 derived from them. It was also noted that both species were more attracted to a group of people rather than to an individual, and biting rates of *C. silacea* increased up to six times as they moved through the forest [31, 52, 55]. *Chrysops silacea* was reported to be more attracted to darker colours or the colour blue/ light blue [72]. In the laboratory, Connal & Connal [22] noted during feeding experiments that guinea pigs with dark patches were bitten more than white ones, and suggested *Chrysops* was able to distinguish colour.

Both *Chrysops* vectors peak biting times were closely associated with the diurnal periodicity of microfilariae of *L. loa* in humans [21, 33]. Several studies in Cameroon, Congo and Nigeria found that these two vectors were almost exclusively active between dawn and dusk [47, 61]. Peak biting times were reported in the morning (*c.*9–11 am), with a decrease around midday and a smaller peak in the afternoon (*c.*3–4 pm) [21, 28, 33, 57, 99, 102]. In Benin, Nigeria labourers were noted to be frequently bitten until midday, when the temperature reaches a maximum and the flies retreated to shaded areas [31]. Detailed studies on *C. silacea* in Kumba indicated that bi-phasic diurnal biting cycle was associated with changes in light intensity, temperature and relative humidity throughout the day. Specifically, the biting activity of *C. silacea* appeared to increase with a rise in temperature to 66–85 °F and decrease with a rise in relative humidity of 56–100% [33, 35].

***Chrysops silacea*** **in forested area** *Seldom attacks in bright sunlight, preferring shade of trees or shelter of verandas, and stops when temperatures reach maximum values in the afternoon. The fly referred to as the ‘softly-softly fly’ as it makes no sound as it hovers. Bites parts not in full view such as back of ankles, legs, outer hands. Bite not painful, but withdrawal is painful, and can cause considerable irritation, extensive swelling for a few minutes to hours after the bites*

#### Host preference and patterns

While *C. silacea* and *C dimidata* were associated with the transmission of human *L. loa*, it was noted that they may attempt to feed on monkeys and other animals during the day; however, with monkeys there was minimal opportunity to take microfilaria from the nocturnally periodic *L. loa* found in monkeys. Host preference studies by Gouteux & Noireau [87] found that both *Chrysops* species had similar feeding patterns and that humans (89–90%) were the main hosts; however, blood meals were also identified from hippopotamus, which were only present in rivers not in close proximity, leading the authors to suggest that *Chrysops* were able to fly over large distances. Gordon et al. [26] raised importance of understanding the relationship between *Chrysops* infective density and human infection rates for control and curative measures, and aimed to defined the different levels of risk, and explain why there may be disparities within and between populations and subgroups such as adults, children, Africans and Europeans.

***Chrysops*** **density, infection and human risk** *… figures of fly-density, fly-infection and an eight-hour biting-period as indicative of conditions at Kumba during the months of June and July, i.e. at the height of the Chrysops season, then, on average, each European would be exposed to the risk of infection with Loa loa once in every five days. (Kumba, Cameroon)*

#### Flight range

Mark-release-recapture studies in Kumba, Cameroon found *Chrysops* could readily travel 1 mile (~1.6 km) in a day, and up to two miles (~3.2 km) through the forest six days after release. In Benin, Nigeria, *Chrysops* were found to fly up to at least 1200 yards (1 km), but this was considered not to be the maximum distance in which the fly could cover [31]. This is in agreement with detailed studies on *C. dimidiata* in Cameroon [94], and another study conducted in secondary forest habitats in Cameroon [97] found the maximum flight range for *C. dimidata* was 4.5 km and for *C. silacea* 2.2 km; however, it was noted that 50% of *Chrysops* were found within 800 m, and 80% within 1500 m from release point.

### Factors influencing spatial-temporal transmission

#### Abundance pattern measures

Adult *Chrysops* abundance was based on biting rates measured as “boy-hours” in historical studies, and by the number of flies caught per man per hour (fly/man/hours) or tabanid per man per day (T/MD) in more recently published articles [91, 99]. Several factors were identified as influencing the biting cycles and infection rates, which were primarily related to spatial and temporal environmental and anthropogenic factors.

#### Spatial environmental factors

Spatial environmental factors were related to the changes in forest density and light intensity both vertically and horizontally. For example, Kettle [35] revealed an association between the diurnal cycle of light intensity measured and the biting cycle of *C. silacea* in Kumba, Cameroon. Further detailed studies of biting and infection rates were conducted at different canopy heights with platforms constructed in the forest for fly-boys to collect species and information on light intensity, temperature and saturation-deficiency [50]. The highest biting and infection rates were found mid- canopy between 28 and 92 ft (~8.5–28 m), which include shaded areas with intermediate light, temperature and saturation measures, compared with the hotter lighter canopy top at 130 ft (~40 m) and the darker cooler ground level sites.

Several studies examined the relationship between forested and cleared areas, and found decreasing biting rates with deforestation related to anthropogenic plantation and human habitation development [90]. However, the rate of reduction varied between sites depending upon the amount and distance from forested vegetation, as well as by species with *C. dimidiata* noted to be more confined to forested areas, e.g. in Makokou, Gabon [101], and in the Chaillu Mountains, Congo [91]. *Chrysops silacea* was more dominant in villages whereas *C. dimidiata* was rarely found in open the environment, favouring primary and secondary forested areas. Duke [53] also examined *C. silacea* differences between a forested site, a total cleared site and a cleared site with rubber saplings. Biting and infection rates measured at regular intervals up to 400 yards (~366 m) in both the cleared sites, showed significant reductions in abundance and infection rates at an increasing distance from the forest site. However, the rates of reduction were more gradual in the cleared site with rubber saplings, compared with the total cleared site).

**Forest clearing and reduction in biting rates** *In a cleared area planted with rubber saplings 10–12 feet high, the biting density fell to one-tenth of the forest value at 530 yards from the forest … In an area of total clearance planted with rubber saplings 1.5–2 feet high, the biting density fell to one-tenth of the forest at 100 years.*

Kershaw [47] also discusses the effect of widespread clearing associated with village, town and commercial development and suggests that strip of half a mile of cleared may be sufficient to significantly reduce human risk.

#### Temporal environmental factors

Temporal environmental factors were related to climate and seasonality. For example, in Kumba, Crewe [57] found that *C. silacea* biting rates increased with rainfall but dropped with the onset of very heavy rain, suggesting that pupae could not survive excessive ground water or flooding. Another study on *C. silacea* in a different part of Cameroon [97], and in the Chaillu Mountains, Congo [91], also found significantly higher biting rates during the rainy season compared with the dry season. Similarly, in areas where *C. dimidiata* was the main vector such as the Cross River State, Nigeria, the highest biting rates were observed during the rainy season, but predominantly late in the season [102]. This late rainy season peak was also noted in Bombe, Cameroon by Duke [54].

#### Wood fires

Wood fires were identified as an additional anthropogenic factor influencing transmission. Duke [43, 51] initially observed that the smoke of wood fires appeared to attract *C. silacea* and detailed studies found a six-fold increase in biting densities of *C. silacea*, but not *C. dimidiata*, in the rainforest in Kumba, Cameroon, with increases most marked during the morning when flies were more common at ground level.

**Wood fire as an attractant** *It is shown that biting density of Chrysops silacea at ground level in the rain-forest at Kumba is increased more than six times when catches are made in the presence of a wood fire. Evidence is produced to show that flies released for biting at canopy level are attracted down to ground level by the smell of wood smoke, thereby accounting for an increased biting density.*

In the Chaillu Mountains, Congo, similar increases in biting densities with the presence of wood fires were found, with a 8.5-fold increase at ground level and 5-fold increase in the canopy for *C. silacea*, but with little or no effect on *C. dimidiata* [93]. More recently Wanji et al. [99] used wood fires as part of the collection tool for a study in Kendonge, Cameroon, recognising it as a field method to increase *Chrysops* numbers for quantification and analysis.

### Methods of vector control

In relation to the control of the *Chrysops* vector, overall few practical measures have been suggested; however, several historical articles referred to studies and potential methods [26, 32, 75–83] of control, which Gordon [28] divided into two main categories and sub-categories including the following: (i) ‛Defensive Methods of Control’: screening and repellents; clearing of forest and of bush; and (ii) ‘Aggressive Methods of Control’: measures directed against adult *Chrysops*; measures directed against immature stages of *Chrysops.*

#### Defensive control measures

Defensive control measures included screening and repellents, which noted several examples, including that in Benin (Nigeria) one house was screened for a period of eight months with no *Chrysops* entering the room, and that 60% or undiluted DMP (dimethyl phthalate) appeared to be a satisfactory personal repellent against *Chrysops,* with protection provided to local workers for a minimum of 2 to 3 h [32]*.* It also included the possible clearing of dense bush in close proximity to housing but concerns were expressed over the practicality of this, and also if it may as a result increase other vectors, such as *Anopheles* and the transmission of malaria [28]. Duke [53] also noted that selective clearing measures may be applicable on organized plantations, where flies are numerous and human populations are at risk in relatively compact areas.

**Screening and repellents for control** *… 60 per cent DMP, when applied to the skin gave complete protection, netting soaked in this solution failed to repel the flies which passed just as readily through the impregnated as through the unimpregnated netting … 30 per cent DMP gives little or no protection against Chrysops.*

**Clearing for control** *… the highest incidence of Chrysops was observed in bungalows lying close to the dense bush. We suggest, therefore, that the annual grant should be increased to allow more generous clearing of bush … since flies appear to approach dwellings along even narrow strips of bush.*

#### Aggressive control methods

Aggressive control methods included those against both the adult and immature stages of *Chrysops* with insecticides*.* For adults, it was suggested that indoor residual spraying (IRS) may help to reduce density as they potentially rest on walls and ceilings waiting to obtain their blood meals, or spraying the undergrowth in the vicinity of the oviposition sites may be of value [28].

For the immature stages, spraying foliage where eggs are laid was suggested, and also the possibility of clearing bush and trees to remove shade or the canalising of streams to remove stagnant vegetation may help to reduce fly density [28, 32]. Detailed studies on the application of DDT (dichlorodiphenyltrichloroethane) dieldrin, aldrin and gamma-BHC (gamma-hexachlorocyclohexane) found that all insecticides were able to penetrate breeding site mud to a depth of 2 to 6 in. (~5–15 cm), with dieldrin most persistent and highly effective as shown in the series of articles on vector control [78, 83]. Williams & Crewe [83] highlighted the success of a 14-square-mile application which reduced *C. silacea* and *C. dimidiata* by 70% and the number of infective larvae of *L. loa* in *Chrysops* by 62%. However, they also noted the difficulties in treating large areas of mud and raised significant concerns about the possible seepage of insecticides into streams, which could create public health problem by adversely affecting other non-target animals and humans. Table 2 further summarises the findings of the studies and discussions highlighted in the article [81].

**Table 2:** Summary of *Chrysops* spp. immature and adult stages, and associated vector control measures

Stage	Target	Aim	Activity	Target area	Category (after Gordon [28])
Larvae	Environmental modification	Reduce or kill pupae/ larval development and emergence	Drainage of water, vegetation clearing to remove shade; flood	Community and surrounds	Defensive
	Insecticide treatment	Reduce or kill pupae/ larval development and emergence	Apply insecticide to mud breeding sites	Community and surrounds	Aggressive
Adult	Personal repellents	Prevent biting by repelling with skin/ clothing impregnated insecticide	Apply insecticide to skin/impregnate clothes of humans	Humans	Defensive
	Household screens, curtains	Prevent indoor biting by a physical barrier/ infrastructure	Wired/meshed windows and doors of houses	Houses	Defensive
	Environmental modification	Reduce abundance by eliminating vegetation/ canopy resting sites	Vegetation clearing around houses/village surrounds	Community and surrounds	Defensive
	Insecticidal treatment	To kill or knock down host seeking	Spraying of foliage near oviposition sites	Community and surrounds	Aggressive
			Indoor residual spraying	Houses	Aggressive
	Traps alone, or with insecticide or wood fire	Reduce abundance by capturing or killing	Proximity to emerging larvae or host seeking	Community and surrounds	Aggressive

**Insecticidal larval spray for control** *Dieldrin emulsion containing one part in 640 of the active agent, applied at the rate of four pints to 100 square feet, kept breeding site free of tabanid larvae for at least eight months. This concentration of dieldrin should be sufficient to control the vectors of loiasis in the rain-forest.*

### Areas of potential future research

Based on the extensive research summarised in this review, the following are considered to be areas of potential future research, which will build on the current knowledge:

(i) Determine alternative trapping methods for collecting adult *Chrysops* spp. that do not involve human-landing catches (i.e. fly-boys);
(ii) Review and assess the potential range of attractants, including wood-fires and trap colour, that may increase adult catch numbers;
(iii) Determine the optimal time and labour efficient methods for identifying breeding sites and collecting larvae for analysis within high risk communities;
(iv) Determine the relationship between *Chrysops* infection rates and human loiasis risk, and if xenomonitoring could play a role in determining the level of risk within a community;
(v) Determine the capacity of local entomologists, community members and field workers to identify main *Chrysops* spp. high risk breeding and biting areas within communities and workplaces to help target control measures;
(vi) Determine if the ecological and climatic aspects of vector habitats and behaviour, including the extent of deforestation and the potential role in reducing risk, can be predicted over larger geographical areas using remote sensing satellite imagery and modelled environmental data;
(vii) Determine the geographical extent of overlapping vector-borne disease infections to better determine how IVM could be effectively implemented.

## Discussion

This paper presents the first extensive review on the two main *L. loa* vectors *C. silacea* and *C. dimidiata* in more than 50 years. This is important as these are neglected vectors of the neglected disease, loiasis, which although not formally listed as an NTD by the WHO has a significant impact on the elimination programmes of LF and onchocerciasis [18]. Studies on the epidemiology of loiasis, and the *Chrysops* vectors that drive transmission should have more prominence as studies highlight the potential clinical impact of loiasis on individuals [8]. Efforts to scale up elimination activities for other co-endemic filarial diseases such as LF and onchocerciasis have been prioritised, and all possible methods of control need to be considered [104]. This review recommends that the control of *L. loa* vectors is considered as an additional strategy to reduce the transmission of *L. loa* where the elimination of LF and onchocerciasis is compromised by the risk of *L. loa* induced encephalopathies; this may be particularly pertinent in hypo-endemic onchocerciasis areas where there are currently no safe chemotherapy options recommended [12], and where currently only doxycycline is a viable alternative chemotherapy [105, 106].

The review highlighted that the majority of studies were conducted in the 1950s and 1960s, when there was a surge of interest in the control of loiasis as an important disease. This was most likely related to the high prevalence found in local populations, rubber plantation workers and palm grove estates. The work from the Helminthiasis Research Scheme in Kumba, Cameroon, and the significant body of related work published in several series of research papers, has provided an important and comprehensive foundation from which to build further work in this field, specifically in relation to the distribution, ecology and epidemiology in high risk areas [2], and methods of targeted vector control, which could be integrated with other vector-borne diseases [107]. However, this will require a further significant surge in interest, funding and purpose for capacity strengthening, as currently there is a general shortage of medical entomologists in Africa, and only a small pool of scientists currently working on *L. loa*.

Moving forward with any form of *Chrysops* control is likely to be multifaceted given that *C. silacea* and *C. dimidiata* are day-biting vectors that breed in densely shaded muddy streams and swamps, and rest in forest canopies high above ground-level. While these characteristics pose significant challenges, several studies indicated that vector control activities can impact on *L. loa* transmission. Therefore, *Chrysops* control or repelling the biting of humans, should be considered as an additional approach to be used in conjunction with other strategies. While this may not be a solution to reducing the risk of SAEs in the short-term given the duration of the transmission cycle, it would provide long-term benefits by reducing the number and intensity of infections, and thereby reducing the frequency of individuals with high Mf loads. The use of modern tools and technology to identify local ‘hotspots’ and initiate vector control/ repellency studies could be successful if targeted at the right place, at the right time, with the right intervention. However, understanding spatial and temporal patterns of the local distributions will be key [108], and not necessary complicated, given that these vectors have readily identifiable physical characteristics, and are primarily associated with forested or plantation areas, with clear seasonality, all of which can be effectively targeted.

For the immature stages of *Chrysops*, the use of community-based environmental management and larviciding with new formulations may be considered. Environmental management including drainage, filling, or removal of vegetation around breeding sites may be possible on a small scale, but is not practical in vast forested areas. The application of insecticide-based larvicides such as temephos (Abate) or biological control agents such as *Bacillus thuringiensis* (Bti) that specifically kill dipteran larvae through regular spraying offers an alternative method. These interventions have low toxicity and have been used widely in Africa for the control of onchocerciasis (*Simulium* spp.), control of *Dracunculus* (guinea worm) intermediate hosts copepods and malaria (*Anopheles* spp.) control [109–112]. The application requires little technical skill, so that community members may be trained to target key sites within vector flight range of 1–2 km, at high risk times based on peak seasonality. Further potential lies with new chemical formulations being developed by the Innovative Vector Control Consortium (IVCC) [113, 114], and innovative field application methods being considered for hard-to-reach places by using smart drones to apply larvicides and adulticides in remote locations using unmanned aerial vehicles (UAV) [115]. However, this approach using UAVs could also focus on the forest edge close to human settlements, to deploy insecticide avoiding the problems of operating within a dense forest environment.

For the *Chrysops* adult stages, the use of personal protection, household screening, IRS, and community-based insecticide spraying or trapping may all help to reduce vector-human contact and transmission. Standard insect repellents have been shown to provide protection to people if applied regularly, especially in the morning peak biting times, however, new methods involving transfluthrin-impregnated hessian strips being trialled against outdoor exposure of malaria (*Anopheles*), urban filariasis (*Culex*) and Zika (*Aedes*) vectors may also be promising for loiasis (*Chrysops*) [116, 117]. Window screening, insecticide-impregnated curtains, and IRS could provide household-level protection, while other innovative community-based approaches such as the blue tiny targets/ traps being used for human African trypanosomiasis (Gambian sleeping sickness) (tsetse) control, may also be capable of reducing transmission by readily placing the targets as key visual stimuli around disease ‘hotspots’ within high risk communities at relatively low cost [118].

These examples also provide insights into the potential for integrated vector management (IVM), with multiple diseases potentially being targeted simultaneously with shared human and financial resource and multiple impact. However, it will be important to first conduct a situational analysis of each disease, including an assessment of the epidemiology and entomology, the extent of geographical overlap, vector control needs and available resources [107]. A systematic review and field assessments of tabanid trapping and control methods in other regions of the world may also help to determine what could realistically be trialled and used in Africa [119–121]. Different trapping methods such as the Nzi trap have been used to monitor species abundance, and attractants such as carbon dioxide (CO_2_) and octanol have shown to potentially improve capture rates, which may be better than the use of wood fires. The development of a trapping-attractant method for the loiasis vectors in Africa could also help with large-scale monitoring. *Chrysops* xenomonitoring has never previously been proposed as tool to determine community risk, but may be a more cost-effective option than labour intensive human seroprevalence surveys or RAPLOA.

Further examination of the current loiasis distribution risk should also be undertaken using the newest remote sensing satellite data sets. Since the initial mapping and remote sensing studies were conducted some 10–15 years ago [2, 4], it is likely that significant deforestation has taken place with human infrastructure development, which will have impacted on the distribution of *Chrysops* in West and Central Africa. It is urgent to utilise remote sensing data to define such risk areas and environmental factors driving transmission, since it is not considered feasible for financial and resource reasons to undertake further RAPLOA studies across such an extensive region, especially in hypo-endemic onchocerciasis ‘hotspots’ [12]. Further, there is a need to better define the areas and extent of risk of SAEs when the implementation of programmes is becoming increasingly urgent if the NTD Roadmap targets are to be met [18].

## Conclusion

This review provides the most recent summary on the current knowledge on the two main *Chrysops* vectors, highlighting main field and laboratory procedures, species distributions, ecology, habitats and potential methods of vector control. Importantly, these factors may help determine the feasibility of how vector control may be implemented to reduce *L. loa* transmission and microfilariae loads in high prevalence communities, and if as a consequence, could also reduce the risk of SAEs associated with the drug ivermectin for LF and onchocerciasis elimination. This is particularly important in areas where high prevalence of *L. loa* are co-endemic with hypo-endemic onchocerciasis ‘hotspots’ and the need for alternative strategies and novel approaches is critical if elimination targets are to achieved. Focussing on those already infected ignores the role that the vector plays in driving the epidemiology and the consequent risk of SAEs.

## Additional files

Additional file 1: *Chrysops* literature database. (XLSX 45 kb)

Additional file 2: Photographs of breeding sites and apparatus for collecting larvae (PDF 626 kb)

Additional file 3: Historical maps of *Chrysops* distributions (PDF 674 kb)

## Abbreviations

APOC: African Programme for Onchocerciasis Control
CDTi: Community-directed treatment with ivermectin
DDT: Dichlorodiphenyltrichloroethane
DEC: Diethylcarbamazine citrate
DRC: Democratic Republic of Congo
GPELF: Global Programme to Eliminate Lymphatic Filariasis
ICT: Immunochromatographic test
IRS: Indoor residual spraying
IVCC: Innovative Vector Control Consortium
IVM: Integrated vector management
LF: Lymphatic filariasis
LLIN: Long-lasting insecticidal net
MDA: Mass drug administration
MF: Microfilaria
NTDs: Neglected tropical diseases
RAPLOA: Rapid Assessment Procedure for Loiasis
SAE: Severe adverse event
UAV: Unmanned aerial vehicles
WHO: World Health Organization
