# Supplementary material for: Loa loa vectors Chrysops spp.: perspectives on research, distribution, bionomics, and implications for elimination of lymphatic filariasis and onchocerciasis
Source: Parasit Vectors. 2017 Apr 5;10:172. doi: 10.1186/s13071-017-2103-y (PMC5382514; doi:10.1186/s13071-017-2103-y)
Supplement: Supplementary file 2 — Photographs of breeding sites and apparatus for collecting larvae (PDF 626 kb) [file 13071_2017_2103_MOESM2_ESM.pdf]

Additional file 2. Photographs of breeding sites and apparatus for collecting larvae

Streams

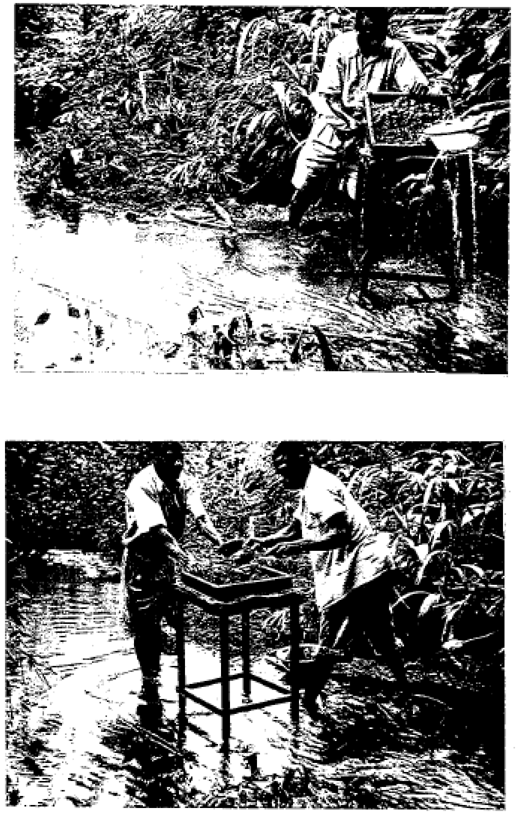

Swamps

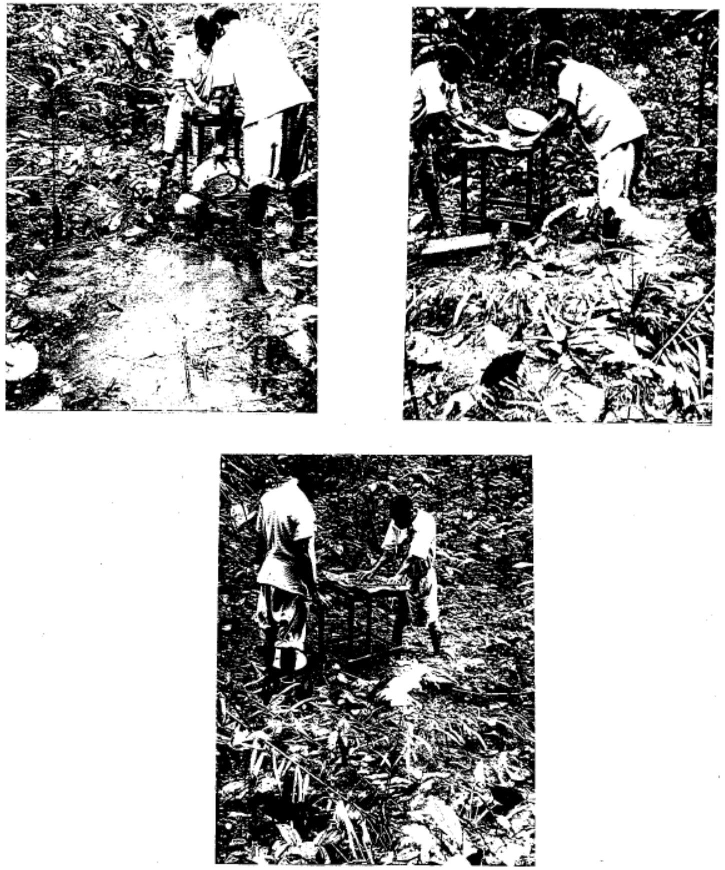

Source: Crewe W. 1956. The bionomics of *Chrysops silacea*, its life history and its role in the transmission of filariasis. PhD Thesis, Liverpool School of Tropical Medicine, Liverpool, UK

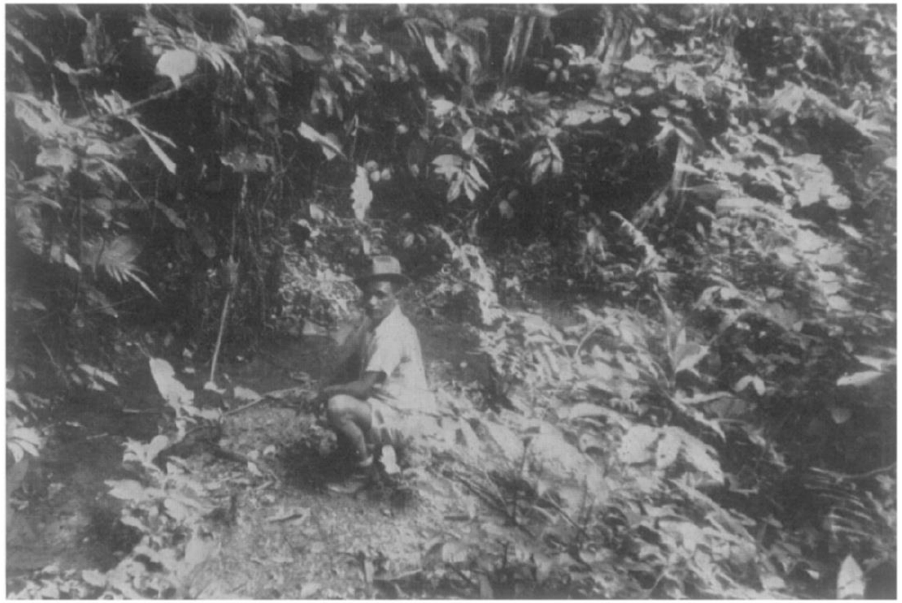

Fig. 1.—View of a typical *Chrysops* breeding place at Kumba. Larvae were found only in streams at points where the flow of water was slow and the bottom covered with sand overlaid with soft mud and decaying leaves. From such sites three species of *Chrysops*, in addition to *C. silacea* and *C. dimidiata*, were bred out, and these included about an equal proportion of male and female flies. (Reproduced, by kind permission, from the *Ann. trop. Med. Parasit.*, Vol. 42, p. 371, Dec., 1948.)

Fig. 2.—View of a typical *Chrysops* breeding place at Sapele. *Chrysops* larvae were found in the streams draining from the borders of rubber plantations into the surrounding mangrove swamps. The sites in which the larvae were found in the streams were precisely similar to those observed at Kumba. So far no adults have been bred from the larvae collected.

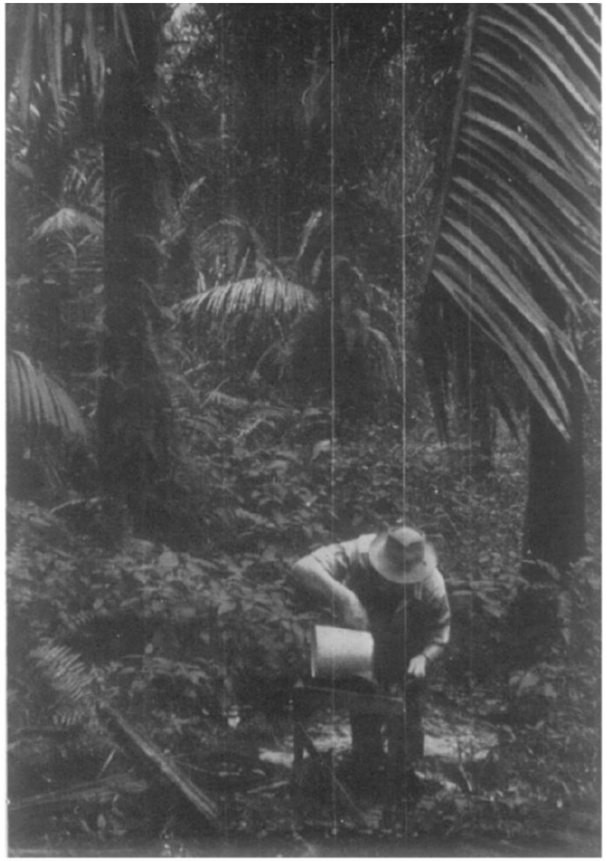

Source: Gordon RM, Kershaw WE, Crewe W, Oldroyd H. The problem of loiasis in West Africa with special reference to recent investigations at Kumba in the British Cameroons and at Sapele in Southern Nigeria. Trans R Soc Trop Med Hyg. 1950;44:11–47
