# Supplementary material for: Loa loa vectors Chrysops spp.: perspectives on research, distribution, bionomics, and implications for elimination of lymphatic filariasis and onchocerciasis
Source: Parasit Vectors. 2017 Apr 5;10:172. doi: 10.1186/s13071-017-2103-y (PMC5382514; doi:10.1186/s13071-017-2103-y)
Supplement: Supplementary file 3 — Historical maps of Chrysops distributions (PDF 674 kb) [file 13071_2017_2103_MOESM3_ESM.pdf]

Additional file 3. Historical maps of *Chrysops* spp. locations

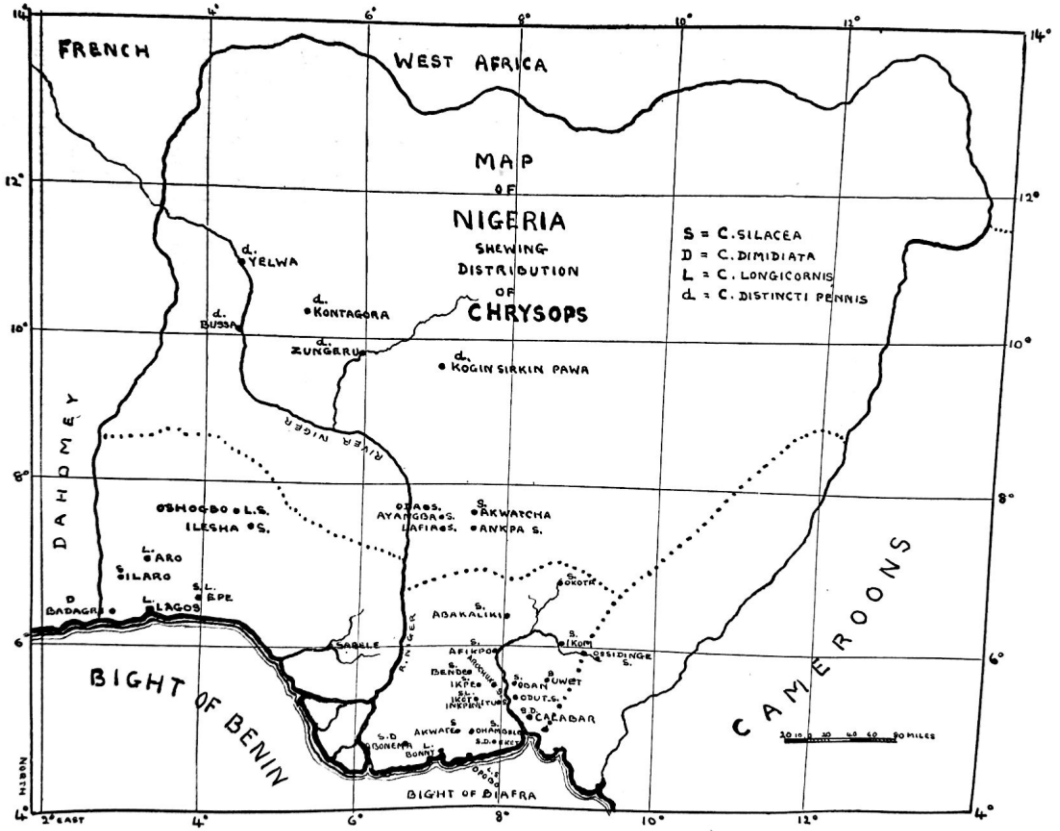

Source: Connal and Connal. 1922. The development of Loa loa (Guyot) Chrysops silacea (Austen) and C. dimidiata (Van der Wulp). Trans. R. Soc. Trop. Med. Hyg. 16: 64-89.

9\. *Chrysops dimidiata* distribution

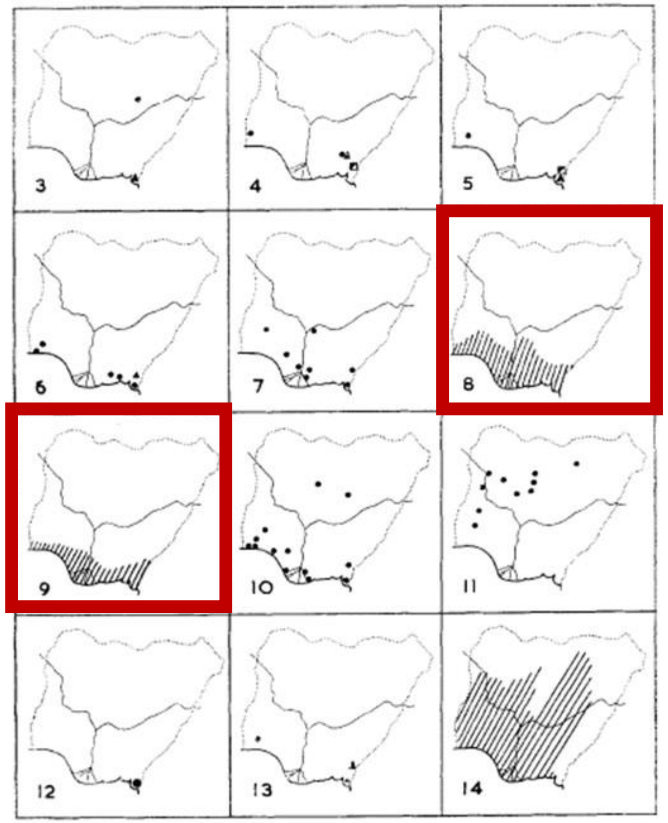

8\. *Chrysops silacea* distribution

MAPS. 3-14.—(3) ●, *Pangonia ruppellii*; ▲, *Dasycompsa cincta*. (4) ●, *Subpangonia gravoti*; ▲, *S. grahami*; ◩, *Nuceria semilivida*. (5) ●, *Thriambeutes singularis*; ▲, *Tabanocella praestabilis*; ◩, *Thriambeutes nigripennis*. (6) ●, *Thaumastocera akwa*; ▲, *Tabanocella schoutedeni*. (7) *Tabanocella stimulans*. (8) *Chrysops silacea*. (9) *Chrysops dimidiata*. (10) *Chrysops longicornis*. (11) *Chrysops distinctipennis*. (12) *Chrysops maximus*, *C. langi*, and *C. centurionis*; these three species are known only from the single locality, Kumba, British Cameroons. (13) ●, *Chrysops griseicollis*; ▲, *C. zahrai*. (14) *Ancala fasciata*.

Source: Crosskey, R.W. & Crosskey, M.E. (1955) The horseflies (Diptera: Tabanidae) of Nigeria and the British Cameroons. Transaction of the Royal Entomological Society of London, 106, 341-374.

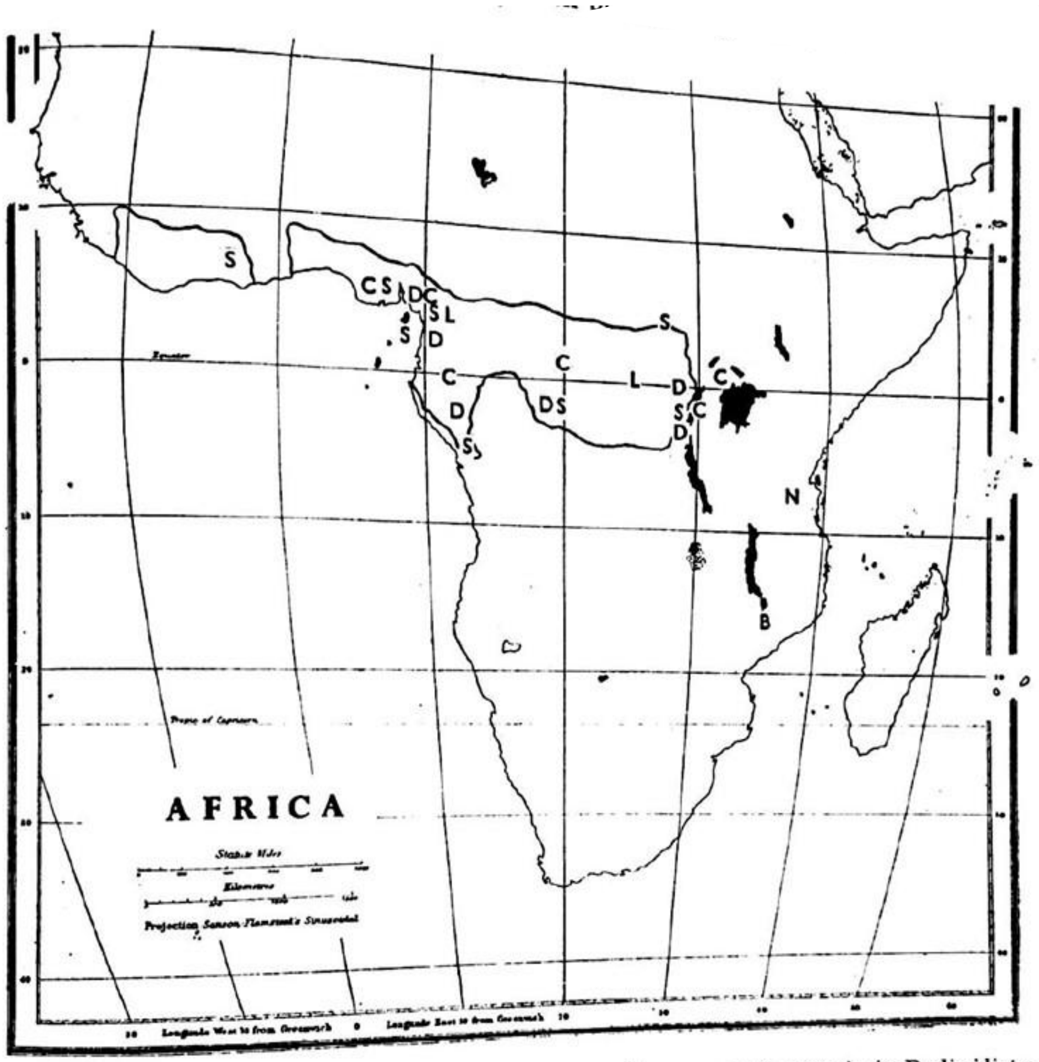

MAP VII. Distribution of the species of the *Chrysops silacea*-group: C, *centurionis*; D, *dimidiata*; S, *silacea*; L, *langi*; B, *bimaculosa*; N, *nigrobasalis*.

Source: Oldroyd H. 1957. Horseflies of Ethiopian Region. Vol 3. Subfamilies Chrysopinae, Scepaidinae and Pangoniinae, and a revised classification. hourse-fly (Diptera Tabanidae) Ethiop. Reg. London: British Museum (Natural History).

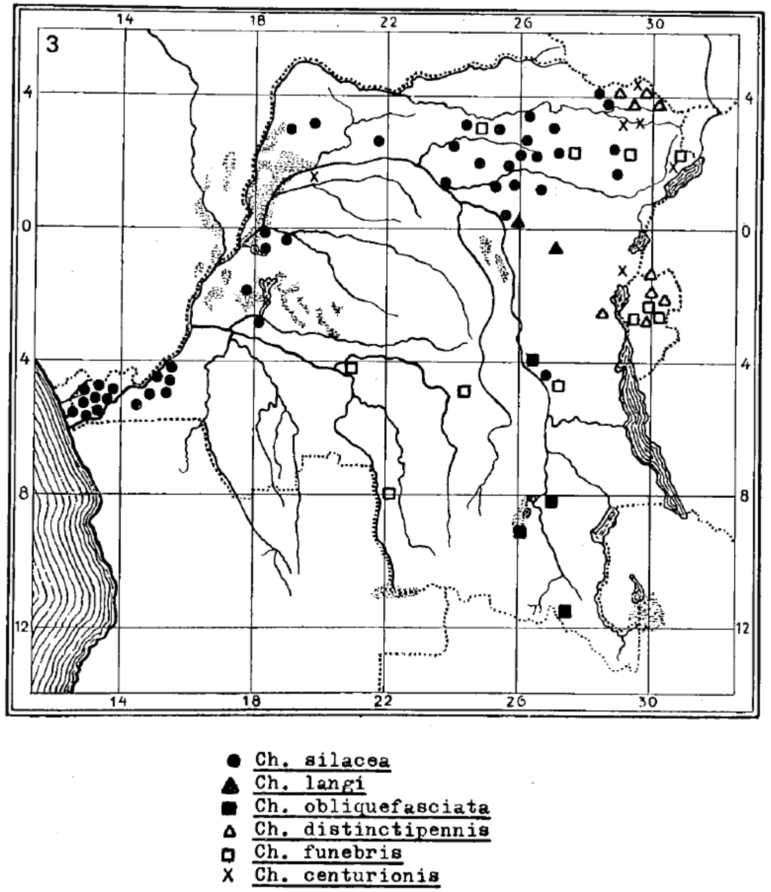

Figure 3
Distribution des *Chrysops* au Congo et au Rwanda.

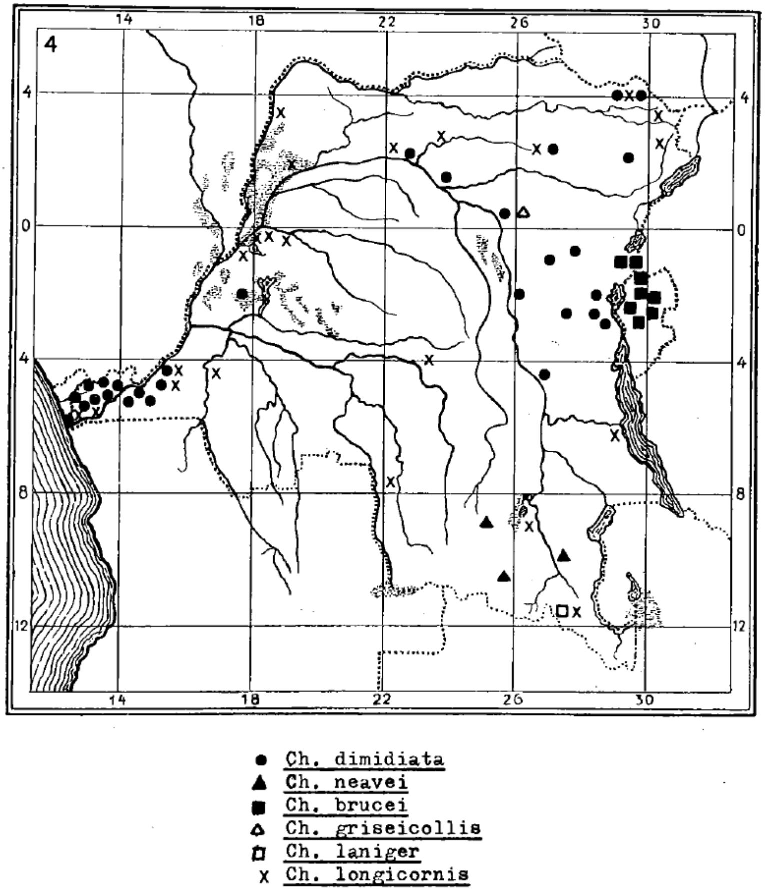

Figure 4
Distribution des *Chrysops* au Congo et au Rwanda.

Source: Fain A. Notes on the geographical distribution of the filaria L. loa and Tabanids of the genus Chrysops in the Congo and in Rwanda. Ann. Soc. Belg. Med. Trop. (1920). 1969. p. 499-530 .
